# Factors Contributing to the False Diagnosis of Misleading Dermatofibromas

**DOI:** 10.1002/cnr2.70164

**Published:** 2025-02-23

**Authors:** Nicholas Florin Kormos, Ioana Daria Paval, Adrian Lucian Baican

**Affiliations:** ^1^ Dermatology Department, Faculty of Medicine “Iuliu Hatieganu” University of Medicine and Pharmacy Cluj‐Napoca Romania

**Keywords:** benign skin neoplasm, clinical diagnosis, dermatofibroma, fibrous histiocytoma, medical topography

## Abstract

**Background and Objectives:**

Dermatofibromas (DFs) are common benign skin lesions with unclear etiology, possibly reactive or tumoral. This study aims to evaluate the incidence of DF misdiagnosis and to correlate histopathological findings with clinical interpretations.

**Methods:**

The extraction for this retrospective study was conducted from 8035 excisions performed between 2016 and 2022 by 9 physicians. A total of 50 cases with conclusive histopathological diagnoses of DF and 33 clinical cases of DF were identified. A *p* value of < 0.05 was considered statistically significant.

**Results:**

The clinical arm included 33 cases, with 7 having DF in the differential diagnosis. The mean age for a confident DF diagnosis was 57.86 years, compared to 44.35 years for a probable diagnosis (*p* = 0.023). Diagnosis accuracy was 69% for certain cases versus 43% for probable cases. DF on legs had higher clinical uncertainty, 6/7 versus 9/26 (*p* = 0.016). In the histopathological arm of 50 patients, lesions on lower extremities had higher correct diagnosis rates (*p* = 0.024) and were least accurate on the torso (*p* = 0.009).

**Conclusions:**

DF can pose a challenge even for experienced clinicians. Location is crucial as DF may escape diagnosis in less common areas. Patients' age and physician's expertise induce uncertainty.

## 1 Introduction

Dermatofibroma (DF) is a common benign skin lesion, characterized mainly by dermal proliferation. It has been defined as (1) a fibrosing cutaneous lesion characterized by an increased number of fibrocytes in the dermis and occasionally subcutis; (2) a variable admixture of macrophages and other inflammatory cells frequently including lymphocytes and rarely eosinophils, neutrophils, and/or plasma cells, with coarse collagen bundles in haphazard array often with peripheral entrapment; and (3) hyperplasia of adjacent structures (epidermis and hair follicles) or cells (melanocytes) [1]. The etiology is debated between a reactive process from a minor trauma or tumoral in nature. A significant proportion of dermatofibromas is associated with previous minor local trauma, especially insect bites. Eruptive lesions have been observed in the context of immunosuppression, HIV infection, highly active antiretroviral therapy (HAART), and pregnancy. Simple excision is usually curative, and local recurrence is rare, generally with rates of less than 2% [2].

Dermatofibroma usually can be found in young and middle‐aged individuals, predominantly in females. It is rather easy to clinically diagnose a DF; however, in certain cases, it can be difficult to differentiate DF from melanomas, dysplastic nevi, or other types of skin tumors [3]. Clinically, DF is characterized as a flat, firm, single or multiple papules, plaques, or nodules, characterized by a range of colors from light to dark brown, or purple‐red to yellow, usually measuring up to 2 cm, with a positive ‘dimpling sign’ [1]. There is a predilection for DF to develop on the lower extremities; however, it can be found anywhere on the body.

The main technique of diagnosing DF includes a clinical evaluation and the use of dermoscopy. The advantages of dermoscopy are its noninvasive characteristic, accessibility, and fast examination [4]. In previous studies, the presence of pigment network and central white patch has been defined as the typical dermoscopic appearance of DF. Nowadays, 4 principal histological structures of DFs are being acknowledged: mainly peripheral delicate pigment network, central white scar‐like patch, white network and homogeneous pigmentation. However, Zaballos et al. identified a total of 11 dermoscopic patterns of DF [5].

Histopathological traits, the most common finding in DFs, are epidermal hyperplasia. Generally, DFs show a dense infiltrate of fibrocytes and/or macrophages in the reticular dermis, with the ratio between these two main types of cells being determined by the stage of evolution of the lesion: in the early stages, macrophages predominate and the late lesions being abundant in fibrocytes and bundles of collagen, frequently arranged in a storiform way [6].

The main histologic variants include fibrocollagenous, cellular, histiocytic, lipidized, angiomatous, aneurysmal, clear cell, monster cell, myxoid, keloidal, palisading, osteoclastic, and epithelioid dermatofibroma. Histological features of several variants can coexist in the same lesion [7]. Immunohistochemical features of DF include epithelioid, lipidized (ankle‐type), myxoid, granular cell, hemosiderotic, keloidal, palisading, atrophic, clear cell, lichenoid, balloon‐cell, signet‐ring cell, and myofibroblast‐rich DF. It can contain intracytoplasmic eosinophilic globules and can be associated with osteoclast‐like giant cells; this classification is important for pathologists to assess a correct diagnosis [8].

The aim of our study is to determine the incidence of DF misdiagnosis and the correlation between histopathological results of DF and the clinical interpretations of those lesions, as well as the main factors that can cause errors in the clinical diagnosis.

## 2 Materials and Methods

The extraction for this single‐center retrospective study was conducted from a pool of 8035 excisions performed by 9 physicians between 2016 and 2022 in the Department of Dermatology at the Cluj County Emergency Clinical Hospital. The study was designed with two arms, with the common inclusion criteria of complete clinical data and histology report; both biopsies and excisions were included. The first arm consisted of cases with a histological diagnosis of dermatofibroma, with the inclusion criteria of a conclusive histological diagnosis of dermatofibroma. The second arm consisted of cases with a clinical diagnosis of dermatofibroma and had the inclusion criteria of a clinical diagnosis or suspicion of dermatofibroma. In total, 50 cases with a conclusive histopathological diagnosis of DF were found that matched the inclusion criteria of the first arm. Another 33 excised lesions had a clinical diagnosis or suspicion of DF, matched the remaining inclusion criteria, and were included in the second arm.

During the clinical examination, a Heine 20 dermatoscope was used to assess and establish the clinical diagnosis or the differential diagnosis of the evaluated lesions. Given the nature of the department's profile, only clinically uncertain or difficult‐to‐manage cases were considered for excision or biopsy, while typical lesions were not excised for cosmetic reasons or at the patient's request. All cases were clinically examined, and histopathological examination was performed to determine a certain diagnosis.

The following clinical features were taken into consideration and analyzed: patients’ personal identification data, age, living environment, date of examination, number of lesions, certain and differential clinical diagnoses, the doctor who performed the examination, and localization. Approval no. 45183/26.11.2024 from the hospital's ethical commission was granted.

The following histopathological features were taken into consideration and analyzed: the performed procedure (biopsy or excision), the dimensions of the lesions (length, width, depth), Breslow index (if appropriate), Clark index (if appropriate), certain and differential diagnoses, whether the clinical and histopathological diagnoses are similar, positive resection margins and their localization if present, and the minimal distance of the closest lateral and deep margin.

A value of *p* < 0.05 was considered statistically significant. The following statistical methods were used: Yates‐corrected Chi‐square test (*χ* ^2^), odds ratio (OR), Fischer's exact test, effect size or Cohen's *d*, and t‐statistic (*t*), and the data were described by the mean value (*M*), standard deviation (SD), standard error of the difference (SEM), 95% confidence interval (95% CI), and the number of cases (*N*).

## 3 Results

The clinical arm of the study contained 33 cases of clinically diagnosed DF and was composed of 14 male cases and 19 female cases. The mean age was 47.2 years, with a slightly lower age mean for women (44.5 years) opposed to males (50.8 years) but without statistical significance (*p* = 0.222). 31 cases were treated as day procedures, and 2 cases were hospitalized patients. A single excision was performed on 21 patients, while 12 patients had two or more excisions. A single biopsy was performed, and the remaining lesions were completely excised.

The clinical diagnosis was considered clinically certain in 27 cases, with 6 cases having uncertain or multiple clinical suspicions. The histological diagnosis was certain in 31 cases. However, 2 cases lacked a certain diagnosis, both being vascular lesions described as possible hemangiomas. The histopathological diagnosis for clinically suspected dermatofibromas was inhomogeneous without a clear pattern (Table 1).

**TABLE 1:** Number of cases in the clinical arm with the differential clinical diagnosis and histopathological diagnosis.

No. of cases	Clinical diagnosis	Histological diagnosis
18	DF	DF
1	DF	Nodular BCC
2	DF	Hemangioma
1	DF	Cutaneous myxoma
1	DF	Solitary neuroma
1	DF	Eccrine spiradenoma
1	DF	Wart
1	DF/nevi	DF
1	DF/In situ SCC	Pilomatrixoma
1	DF/Lipoma	Prurigo nodularis
1	DF/Trichilemmoma	Large cell acanthoma
1	DF/Epidermal cyst	Hypertrophic scar
1	DF/Melanoma	DF
1	Unknown, probably DF	DF

Abbreviations: BCC, basal cell carcinoma; DF, dermatofibroma; SCC, squamous cell carcinoma.

Age played a key error factor in clinical diagnosis. The mean value for the certain diagnosis group (*M* = 44.59, SD = 13.79, SEM = 2.65, *N* = 27) was significantly lower than the mean value for the group with an uncertain diagnosis (*M* = 59.00, SD = 10.51, SEM = 4.29, *N* = 6), a mean difference of 14.41, 95% CI [4.52, 24.30]. This difference was statistically significant, *t*(9.30) = 2.86, *p* = 0.018, with a large effect size, Cohen's *d* = 1.08. There was no significant difference between the correlation of the clinical and histological diagnosis among the certain and uncertainly diagnosed cases, with 18/27 (67%) for the group with a certain diagnosis and 3/6 (50%) for cases with a suspicion of DF (*p* = 0.241).

Another error factor was the excision site, which was consistent in determining the certainty level of a clinical diagnosis (Table 2). DFs located on the lower extremities produced more clinical uncertainty in the diagnosis. 15/33 (45%) of all lesions were located on the lower extremities, while 6/7 (86%) of uncertain lesions were located on the lower extremities, with a significant difference *χ* ^2^ (1, *N* = 33) = 3.93, *p* = 0.047, OR = 0.088 [0.01, 0.85], and Fisher's exact test confirmed the association between the variables (*p* = 0.03).

**TABLE 2:** Location of excised DF and their clinical diagnosis certainty level.

	Certain diagnosis	Uncertain diagnosis	*p*
Head and neck	2	1	> 0.99 (Fisher's exact)
Lower extremities	9	6	0.047
Upper extremities	12	0	—
Torso	3	0	—
Total	26	7	

The histopathological arm of the study had 50 cases histologically diagnosed as DF, of which 20 were males and 30 were females (2:3 ratio), a mean age of 49.2 years, with a slightly higher mean age for women (50.0 years) opposed to males (47.9 years), without significance (*p* = 0.593). 46 cases were treated as day procedures, and 4 cases were hospitalized patients. A single excision was performed on 35 patients, with the remaining cases having two or more excisions performed. 6 biopsies were performed, and the remaining lesions were completely excised. The lesions that were biopsied had a clinical diagnosis of basal cell carcinoma (BCC)—3 cases: DF, unspecified vasculitis, and keratoacanthoma.

The clinical diagnosis was considered certain in 26 (52%) cases, with 24 (48%) cases having uncertain or multiple clinical suspicions. The overall diagnostic accuracy for DF was 24/50 (48%), with 26/50 (52%) having different clinical suspicions (Table 3). The histological diagnosis was certain in 47 cases, and for 3 cases, DF was considered to be the most likely differential diagnosis. For one case, immunohistochemistry was not available; another case could not fully exclude dermatofibrosarcoma, and the last one had a differential diagnosis with a scar tissue.

**TABLE 3:** Number of cases in the histopathological arm with the differential clinical diagnosis and stratification based on clinical diagnosis certainty.

No. of cases	Clinical diagnosis	Certain diagnosis	Unsure diagnosis
24	DF	16 (66%)	8 (33%)
6	BCC	2 (33%)	4 (66%)
5	Nevi	3 (60%)	2 (40%)
2	DF/melanoma	0 (0%)	2 (100%)
2	DF/nevi	0 (0%)	2 (100%)
2	Epidermal cyst	0 (0%)	2 (100%)
1	Epidermal cyst/breast cancer metastasis	0 (0%)	1 (100%)
1	Seborrheic keratosis	1 (100%)	0 (0%)
1	Acrochordon	1 (100%)	0 (0%)
1	Glomangioma	0 (0%)	1 (100%)
1	Keratoacanthoma	1 (100%)	0 (0%)
1	Lipoma	1 (100%)	0 (0%)
1	Unspecified benign tumor	0 (0%)	1 (100%)
1	Bednar tumor	0 (0%)	1 (100%)
1	Vasculitis	1 (100%)	0 (0%)
50	TOTAL	26	24

Abbreviation: BCC, basal cell carcinoma.

The highest correlation between clinical and histopathological diagnosis was observed in lesions located on the lower extremities with the *χ* ^2^ (1, *N* = 50) = 3.88, *p* = 0.048, OR = 3.93 [1.17, 13.28], and the association was confirmed by Fisher's exact test (*p* = 0.047) (Table 4). Out of those correctly diagnosed, 0/3 (0%) cases were located on the head and neck area, 13/19 (68%) cases on the arms and shoulders, 8/13 cases (62%) on the lower extremities, and 3/15 (20%) cases on the torso. Lesions on the torso presented the highest diagnostic difficulty with *χ* ^2^ (1, *N* = 50) = 5.22, *p* = 0.022, OR = 6 [1.42, 37.74], and the association was confirmed by Fisher's exact test (*p* = 0.02).

**TABLE 4:** Location of lesions from the histopathological arm and number of correct diagnoses dependent on lesion location.

Location	Correct diagnosis	False diagnosis	*p*
Head and neck	0	3	—
Lower extremities	13	6	0.024
Upper extremities	8	5	0.256
Torso	3	12	0.048
Total	24	26	

Clinician's experience was a source of bias for diagnosed lesions. One physician had a 4/4 (100%) correct diagnosis of DFs located on the upper extremities but 0/2 (0%) of lesions located in other areas. Another well‐experienced physician had 0/3 (0%) correct diagnosis. Meanwhile, a third physician achieved an 11/13 (85%) correct diagnosis rate for DFs located on extremities. The remaining 6 physicians obtained results consistent with those obtained by the group as a whole and did not present any other predictive factors that were not already analyzed (Table 5).

**TABLE 5:** All analyzed risk factors with their influence on the clinical diagnosis of dermatofibromas.

Analyzed factor	Influence	OR, 95% CI	*p*
Age	Younger patients were more likely to have a certain diagnosis than an uncertain one	14.41 years [4.52, 24.30]	0.018
Location	Lower extremities—induced clinical uncertainty	0.088 [0.01, 0.85]	0.030
	Torso—histologically confirmed DF had poor correlation with clinical diagnosis	6 [1.42, 37.74]	0.020
Clinician experience	Diagnosis accuracy can vary widely from 0% to 100%	—	—
Diagnosis certainty	Lesions certainly diagnosed on the lower extremities were more accurately diagnosed	3.93 [1.17, 13.28]	0.047
Age difference	No difference among histologically confirmed DF	—	0.593
Clinical certainty	No difference in diagnosis when the clinical diagnosis is certain versus uncertain	—	0.241

Abbreviations: DF, dermatofibroma; OR, odds ratio and two standard deviation intervals; *p*, probability value.

## 4 Discussion

DFs are usually an easy diagnosis for dermatologists, and dermoscopy made the diagnosis even easier to perform. Even so, the diagnosis may be challenging and require a biopsy or a complete excision. Difficulties in correctly diagnosing DF can arise from possible collisions with other benign or malignant tumors [3, 9]. It is worth noting that our cohort of cases included only difficult‐toto‐diagnose DFs, where the differential diagnosis was challenging for clinicians.

From our cohort of histopathologically confirmed DFs, less than half were diagnosed solely as DF, which is conclusive with other reported cases or case series regarding atypical DF or rare histological variants with challenging clinical presentations [9, 10]. Similar to the two cases in our cohort, there are instances where the clinical presentation can be that of a melanoma; this raises the prospect of an immediate biopsy or excision [11]. Even though dermoscopy can aid in increasing the number of correctly diagnosed DF, the challenging aspect of rare presentations still remains, and probably, new diagnostic tools like confocal microscopy could make changes in the approach to such cases [3, 5].

Although dermatofibromas are considered more akin to younger patients and malignant lesions are usually considered to be a characteristic of older patients, each lesion should be individually diagnosed based on clinical presentation [1, 3, 8]. As age can be a misleading factor even for experienced clinicians, an age‐related bias may increase the number of unwanted excisions and induce biopsy fatigue [12]. The cases analyzed in this study stressed the implications of age in clinical certainty. This has further implications both from a patient‐centric approach and also from a healthcare perspective. Unnecessary surgical procedures can lead to risks and further avoidable complications and also a certain rise in healthcare costs for patients and healthcare systems [13]. Teledermatology can have a crucial role in correctly assessing the malignancy of lesions in the general population. The use of teledermoscopy in such situations can be especially beneficial when considering difficult‐toto‐diagnose lesions [14].

Another pitfall of benign lesions is the resemblance to other noncutaneous tumors. Mitsui et al. presented a case of breast carcinoma mimicking DF; this can go both ways as one of the cases included was initially suspected of being a cutaneous metastasis of prostate cancer [15]. Another case by Kinoshita et al. described a giant DF. Such cases may pose a significant challenge and require extensive investigations to establish the correct diagnosis of a benign tumor and to infirm the presence of malignancy [16, 17]. Atypical Spitz tumors can also represent such a challenge as they pose a similar risk of being falsely diagnosed with more severe lesions as some dermatofibromas [18]. The implications can be severe, especially when histological confirmation is delayed and clinicians may opt for a more aggressive response than required, which could further decrease the quality of care [19].

Most DFs are usually located on the lower extremities, although they can be localized anywhere on the body [1]. The cohort arm of histologically confirmed DFs revealed an almost equal distribution of lesions on the lower and upper extremities and torso; however, clinicians were more confident in diagnosing lesions on the lower extremities as DF. Even though lesions that were clinically uncertain were more likely to have a misdiagnosis. No lesion from the head and neck area was correctly diagnosed clinically, and just 3/15 lesions on the torso were correctly diagnosed as DF. Alves et al. described the correlation between the histopathological differences among the different body areas and their correlation with clinical variants. This, in turn, may explain why, in our cohort, clinicians were more inclined to diagnose lesions on the lower extremities [2]. Brancaccio et al. described the presence of a different dermoscopic pattern for DF on the trunk compared to the extremities, with a different pattern than those of DFs on the extremities, which further explains the difference between the results observed in our cohort [20]. Both arms of our cohort presented with significant variations and mismatches in diagnosis; this underlines the challenge in obtaining a correlation between the clinical and histological diagnosis [21, 22].

Considering the differential diagnosis posed with malignant tumors, such as melanoma, squamous cell carcinoma, and basal cell carcinoma, the necessity of biopsy or excision is paramount. Even in esthetically sensitive areas, biopsies should be performed as clinical melanoma‐like features have been reported, and the exclusion of a melanoma must be performed. AlJasser et al. described the uncertainty around conclusively differentiating DFs from melanoma by using dermoscopy even when regarding the usual findings either in cutaneous melanoma or in DFs [23]. Ferrari et al. published even higher numbers of clinical resemblance than in our cohort, as they reported melanoma patterns in 16.2% of DFs examined compared to our 4%, but lower rates of basal cell carcinoma of just 3.8% against our reported 12% [24].

Personal bias and clinician expertise seem to play a role in the management and diagnosis of DFs as the rate of correct diagnosis in our cohort ranged from 0% to 85%. It can be assumed that a low number of excised or biopsied DFs and the false diagnosis were in line with the physician's certainty of a completely different skin lesion. Likewise, in the case of the physician with the highest rate of correct diagnoses, it can be assumed that the diagnosis of DF was taken as a clinical certainty; however, probably, a more severe diagnosis could not be excluded.

A correct diagnosis is essential in clinical practice, both for the patient and the practitioner. An accurate clinical diagnosis results in a decided therapeutic management, which, in turn, can promote a faster case management regardless of whether the lesion is benign or malignant. A 2007 study conducted in Australia regarding melanoma screening programs performed by either a general practitioner or a dermatology specialist reported a mean cost reduction per diagnosis and early management per patient of 118–128 AUD. The mean cost savings were greater for melanoma, squamous cell carcinoma, or basal cell carcinoma than for benign conditions [25]. Therefore, a better diagnosis of dermatofibroma can reduce costs as the treatment procedures, and adjacent costs are significantly lower.

Another study conducted by Shir‐Az et al. analyzed the cost reduction of dermoscopy in screening cutaneous lesions and determined a significant accuracy increase and a cost reduction. By using dermoscopy alone, a total of 35.392 excisions could have been prevented in Israel in 2011 and would have saved up to 5 127 532 USD in unnecessary costs regarding surgical procedures and histological examinations [26].

There are a few limitations to this study. First, the number of excised DFs from our cohort did not represent all cases, as plenty of patients had a certain clinical diagnosis of DF, and the lesion was neither excised nor biopsied. Therefore, this study is representative only for DFs that are already challenging and pose a significant risk for differential diagnoses. A consequence of this was the relatively low number of included cases, which diluted the statistical strength of the study.

The setting of the study was a teaching hospital; therefore, there was a wide range of physician experience levels and clinical diagnostics criteria applied. The study is highly dependent on the clinical data and diagnostics accuracy; however, considering that a limited number of physicians evaluated most cases within the same institution, the method of reporting was similar, and a broad variance in diagnostic skills was present, which may have been similar to that experienced in other settings.

## 5 Conclusion

Dermatofibromas are usually easy to recognize; however, in specific instances, the diagnosis can represent a challenge. The diagnosis of some DFs may pose a challenge even when considering the current advantages clinicians have, and there are several factors which may impede the success rate of correctly diagnosing a DF. Age, location, and physician experience were the main groups of factors that were found to have an influence on clinically diagnosing DFs. These factors can mislead clinicians into diagnosing a benign condition as an aggressive form of cancer, which can lead to unnecessary interventions, patient anxiety, and, in rare instances, significant morbidity. Dermoscopy is the most accessible tool for clinicians and can significantly improve the diagnosis rate of clinicians. Histology is still the gold standard, and it can, in most instances, establish a certain diagnosis. Although the clinical presentation and dermoscopy may not be enough, performing a biopsy would significantly increase the likelihood of a correct diagnosis and decrease the chances of comorbidities of unnecessary procedures, especially in elderly patients and challenging locations. Further multicentric studies with larger numbers of cases could provide more insight.

## Author Contributions

**Nicholas Florin Kormos:** conceptualization (equal), data curation (equal), investigation (equal), methodology (equal), project administration (equal), validation (equal), writing – original draft (equal), writing – review and editing (equal). **Ioana Daria Paval:** data curation (equal), formal analysis (equal), investigation (equal), validation (equal), writing – original draft (equal), writing – review and editing (equal). **Adrian Lucian Baican:** conceptualization (equal), data curation (equal), formal analysis (equal), investigation (equal), methodology (equal), project administration (equal), supervision (equal), writing – original draft (equal), writing – review and editing (equal).

## Conflicts of Interest

The authors declare no conflicts of interest.

## Data Availability

The data that support the findings of this study are available from the corresponding author upon reasonable request.
